# TGFβ/BMP immune signaling affects abundance and function of *C. elegans* gut commensals

**DOI:** 10.1038/s41467-019-08379-8

**Published:** 2019-02-05

**Authors:** Maureen Berg, David Monnin, Juhyun Cho, Lydia Nelson, Alex Crits-Christoph, Michael Shapira

**Affiliations:** 1 Department of Integrative Biology, University of California, Berkeley, CA 94720 USA; 2 Graduate Group in Microbiology, University of California, Berkeley, CA 94720 USA

## Abstract

The gut microbiota contributes to host health and fitness, and imbalances in its composition are associated with pathology. However, what shapes microbiota composition is not clear, in particular the role of genetic factors. Previous work in *Caenorhabditis elegans* defined a characteristic worm gut microbiota significantly influenced by host genetics. The current work explores the role of central regulators of host immunity and stress resistance, employing qPCR and CFU counts to measure abundance of core microbiota taxa in mutants raised on synthetic communities of previously-isolated worm gut commensals. This revealed a bloom, specifically of *Enterobacter* species, in immune-compromised TGFβ/BMP mutants. Imaging of fluorescently labeled *Enterobacter* showed that TGFβ/BMP-exerted control operated primarily in the anterior gut and depended on multi-tissue contributions. *Enterobacter* commensals are common in the worm gut, contributing to infection resistance. However, disruption of TGFβ/BMP signaling turned a normally beneficial *Enterobacter* commensal to pathogenic. These results demonstrate specificity in gene-microbe interactions underlying gut microbial homeostasis and highlight the pathogenic potential of their disruption.

## Abstract (web summary)

Host genetics modulates gut microbiota composition, which in turn affects host health, but little is known about the specific genes involved. Here, Berg et al. show that TGFβ/BMP immune signalling affects abundance and function of commensal *Enterobacter* species in *C. elegans*.

## Introduction

All animals harbor complex communities made of diverse microbes, and those of the gut are the most extensive ones. Gut microbes are often referred to as commensals—that is, causing no harm and having no benefit—and in any given condition some may indeed be just so, but overall, gut microbiotas are beneficial, contributing to features as diverse as development, metabolism, immunity, fecundity, and even behavior^1–5^. Furthermore, abnormal microbiota composition (or dysbiosis) is associated with pathology, and in some cases (i.e., obesity and potentially aging) has been shown to play causal roles^6,7^.

In determining the factors that shape microbiota composition, work in vertebrates has been instrumental in revealing a significant impact of diet^7,8^. Environmental factors, such as geography, or life style, were also shown to contribute^9–11^. Less is known about the role of genetic factors, which was suggested to have a relatively modest effect size on the microbiota^12^. Nevertheless, one might expect that advantages provided by beneficial microbes to a host over its peers should promote selection of genes and gene variants that enable colonization by such microbes, resulting in host-specific microbiotas shaped to varying degrees by genetic factors. Consistent with this, species-specific gut microbiotas have been identified in various organisms, including apes, bees, termites, and *Caenorhabditis elegans*^13–16^. In a few instances, composition of these microbiotas was shown to be associated with host evolution. For example, in bees, the appearance of specific core gut bacterial lineages coincides with the emergence of eusocial bees from solitary ancestors^14^, and the composition and functional impact of microbiotas was found to track phylogenetic relatedness between species of several clades, including deer mice, *Drosophila*, mosquitoes, and wasps^17^. Our own analysis of nematode microbiotas identified a significant contribution of host genetics to microbiota composition^18^. However, the specific genes behind such contributions are mostly unknown. Results from human studies demonstrated that host metabolism and immunity can shape the human gut microbiota. Human twin studies identified the lactase gene locus as associated with the abundance of *Bifidobacterium*^19^, and innate immune genes, such as C-type lectins, have been shown to contribute to shaping human gut microbiota function and composition^20^. Whereas human studies rely on associations between natural genetic variation and microbiome composition, studies using model organisms, such as mice, can directly test the effect of a specific host function. In one such study, mice lacking *CARD9*, an adaptor protein required for innate immune responses, were found to harbor an altered microbiota, compromised in the production of aryl hydrocarbon receptor ligands, which led to increased susceptibility to colitis^21^. However, for the most part, distinguishing gene effects from inter-individual variation in vertebrate models is not trivial, hindering the ability to identify influences of host factors on microbiota composition.

An alternative is offered by invertebrate models such as *Drosophila melanogaster* and *C. elegans*. Studies in *Drosophila* have identified mechanisms enabling immune tolerance of gut microbes, and determining the abundance of gut commensals^22,23^. *C. elegans* offers the additional advantage of working with self-fertilizing genetically homogeneous populations, averaging-out inter-individual variation to discern gene effects. *C. elegans* has been used extensively for studying molecular mechanisms of innate immunity^24,25^, but decades of growth on monoxenic cultures, typically of an *Escherichia coli* strain unable to colonize healthy worms, has left a gap in the understanding of its biology and its interactions with benign microbes. This is now changing. Studies of *C. elegans* interactions with different food bacteria provide insights into metabolic regulation and aging^26–29^, and recent work defined a characteristic *C. elegans* gut microbiota, and showed that its composition was conserved across different strains and geographical locations^13,30,31^. Moreover, this composition bore functional significance for worms, with positive impact mainly on development and on immunity, provided typically by *Pseudomonadaceae* and *Enterobacteriaceae* bacteria, including host-specific contributions (reviewed in^32^).

Taking advantage of the availability of *C. elegans* mutants, we examined the contribution of host genes to shaping the *C. elegans* gut microbiota. RNAseq identified genes involved in digestion and in innate immunity as those upregulated during interactions with complex microbiotas. Analysis of mutants for genes central to these processes, using synthetic communities composed of previously isolated worm gut commensals, and providing a defined environment, identified a role for Transforming Growth Factor (TGF)β/Bone Morphogenetic Protein (BMP) signaling in controlling bacterial abundance of *Enterobacter* commensals and in determining their contributions to the host.

## Results

### Genes modulated during interactions with complex microbiotas

RNAseq analysis was performed to identify *C. elegans* genes and processes involved in host–microbiota interactions, comparing gene expression in worms grown on complex environmental microbiotas to that in worms grown on *E. coli*. Two comparisons were performed: one in composted soil microcosms (autoclaved compost reconstituted either with the microbiota from an unautoclaved batch of the same soil, or with a saturated *E. coli* culture); the second on plates, seeded either with *E. coli* or with synthetic microbiotas prepared with equal portions of 30 *C. elegans* gut isolates representing the main core microbiota families (SC1, see Methods). Analyses were performed in age-matched adult worms from synchronized populations; three independent populations were analyzed per group. Measurements were obtained for 28,555 unique RNA transcripts (measured in at least one sample), representing 18,873 genes (see Data availability).

**Fig. 1:**
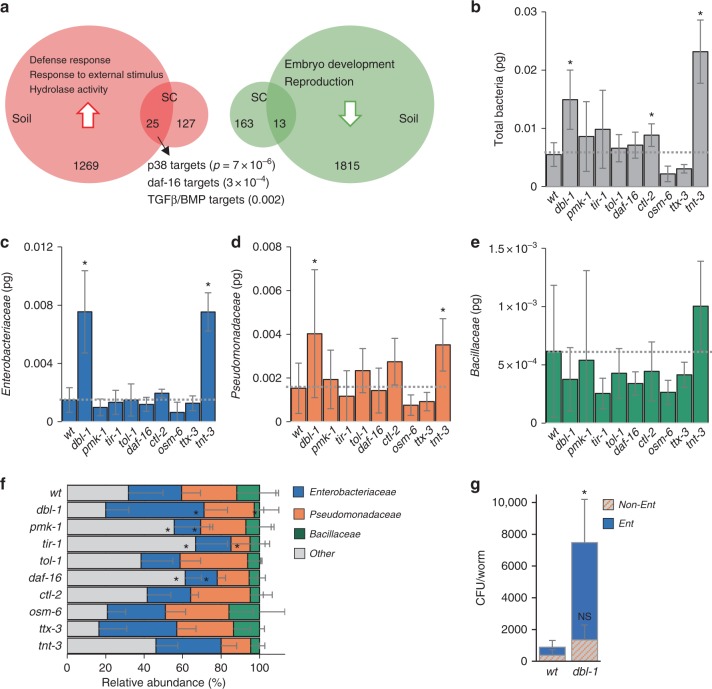
Genes affected by host–microbiota interactions and involved in shaping the gut microbiota. **a** Numbers of, and annotations enriched among, genes differentially expressed in worms raised on complex environmental microbiotas compared with those raised on *E. coli* (detailed in Supplementary Data files 1 and 2 and Supplementary Table 1). **b**–**e** Bacterial load in worms of the designated strains raised on the SC1 community (in pg 16S rDNA, see Methods): **b** All Eubacteria, **c** *Enterobacteriaceae*, **d** *Pseudomonadaceae*, **e** *Bacillaceae*. Shown are averages ± SD of 2–4 independent experiments. Measurements were performed on a pool of 30 worms per experiment. **f** Relative abundances of each group were calculated based on the values shown in **a**–**d**; light gray bars represent relative abundance of all ‘other’ bacterial groups not directly measured. *p < 0.05 (analysis of variance (ANOVA)) compared with N2. **g** Colony-forming units (CFUs) representing live bacteria extracted from worm guts, cultured and counted on *Enterobacteriaceae*-selective media plates (Ent) or on rich media plates (LB), which following subtraction of Ent stands for non-*Enterobacteriaceae* bacteria. Shown are averages ± SD of counts from four plates (*n* = 10 worms) per group from a representative experiment of four showing similar results. *p = 0.006, *t*-test

In worms raised on the synthetic community, 127 genes were significantly upregulated and 163 genes were significantly downregulated compared with worms raised on *E. coli* (false-discovery-rate-corrected *q*-value < 0.05, likelihood ratio test, Supplementary Data file 1). Enrichment was found among the upregulated genes for immune genes, as well as for hydrolases (peptidases in particular) (Fig. 1a, Supplementary Data file 2). This is in spite of lack of any indication that SC1 included pathogens that compromised worm survival (Supplementary Fig. 1a), suggesting that elevated immune activity underlies normal host–microbiota interactions. Among the downregulated genes, only broad gene ontology (GO) terms, such as catabolic processes, were enriched.

Many more genes were affected in worms raised in composted soil microcosms: 1269 genes were significantly upregulated and 1815 downregulated, compared with worms grown on *E. coli*-supplemented soil (Supplementary Data file 1). The larger number of differentially expressed genes in soil-grown worms compared with those raised on SC1 indicates that *C. elegans* does not respond to complex microbiotas in a stereotypical way and suggests that the extent of changes in gene expression may depend on microbial diversity. Among both upregulated and downregulated genes, we found enrichment for genes associated with developmental programs, and to a lesser degree (and specific for downregulated genes) with reproduction (Supplementary Data file 2). In agreement with this, gravid worms harvested from normal soil microcosms held fewer eggs in their uterus compared with those raised on *E. coli*, either on plates or in soil (one row of eggs versus two). Using SC1 plates, we followed worm development and reproduction more closely. Worms raised on SC1 started laying eggs 1–2 h earlier than worms raised on *E. coli*, but laid in total 25% fewer eggs (Supplementary Fig. 1b). This correlated with the identified expression trends, and suggested that exposure to a diverse microbiota modulated host development resulting in a trade-off between development and fecundity. Another prominent trend emerging in microcosm-raised worms was enrichment for upregulated genes involved in response to external stimuli, many of which were immune genes (*p* = 5E–15, Bonferroni corrected, Supplementary Data file 2). This corroborated the trends observed in worms raised on SC1.

The overlap between genes significantly upregulated on microcosm microbiotas or on SC1 included 25 genes (Table 1). The great majority of these (21/25) are reported to be expressed in the intestine (Wormbase [http://www.wormbase.org]). These included C-type lectins, saposins, peptidases, as well as enzymes involved in sphingomyelin metabolism, and were significantly enriched for genes associated with defense and immune responses (*p* = 6E–5, Bonferroni corrected) and for genes encoding hydrolytic enzymes (*p* = 4E–5), including several lysosomal enzymes (*p* = 2E–6), pointing to immune functions as a common denominator among host factors that interact with the microbiota. Expression patterns for several of the genes were confirmed by quantitative (q)RT-PCR, see Supplementary Fig. 2.

**Table 1:** Genes upregulated during interactions with complex microbiotas

Gene	SC1/*E. coli*^a^	*q*-Val	Soil/*E. coli*	*q*-Val	Concise description^b^
Y65B4BR.1	48.4	1.8E–22	6.4	9.3E–24	Defense response; predicted hydrolase
F53A9.8^c^	11.3	2.4E–14	5.2	1.4E–08	Defense response to G + bacteria; intestinal
clec-52^c^	13.5	1.2E–11	12.7	1.2E–25	A Collectin homolog; defense response to G + bacteria; expressed in pharynx, neurons, intestine
asah-1^c^	4.8	2.4E–09	6.4	3.5E–100	*N*-acylsphingosine amidohydrolase 1 ortholog; determination of adult lifespan; intestinal
C32H11.4^c^	30.9	2.8E–09	5.1	1.6E–04	Epoxide hydrolase 1 ortholog; immune response
asp-14^c^	7.8	4.4E–09	2.0	2.1E–03	Encodes an aspartyl protease
asah-2^c^	1.8	1.3E–05	4.3	4.0E–39	*asah-1* paralog; enriched in: intestine, germ line, PVD/OLL neurons
clec-65^c^	3.4	1.4E–05	1.3	1.4E–02	Expressed in the intestine
spp-8^c^	1.9	3.3E–05	8.8	6.0E–44	Prosaposin-like 1 ortholog; enriched in: intestine, germ line, PVD/OLL neurons
scl-2^c^	1.8	2.6E–04	4.1	9.1E–21	A SCP/TAPS-domain protein. Expressed in the intestine
spp-18^c^	2.6	3.2E–04	4.0	1.3E–15	Innate immune response
hpo-15^c^	9.1	7.8E–04	3.6	2.7E–09	Polyamine oxidase (exo-N4-amino) ortholog; determination of adult lifespan
C14C6.2^c^	2.2	2.0E–03	2.2	4.8E–02	Enriched in: intestine, PVD/OLL neurons
irg-3^c^	13.3	2.0E–03	3.6	3.6E–04	Innate immune response
clec-84^c^	2.0	2.4E–03	2.0	4.8E–03	Enriched in: intestine, pharyngeal muscle, germ line
lips-15	3.2	4.3E–03	5.3	4.7E–04	Predicted hydrolase
cyp-37B1^c^	4.3	4.4E–03	2.6	4.0E–02	Cyt P450 homolog; defense response to G + ; intestinal
cpr-3^c^	4.6	1.0E–02	8.7	3.9E–12	Cathepsin B ortholog; involved in embryo development, immune response; expressed: pharynx, pharyngeal-intestinal valve, intestine, rectal gland
nuc-1^c^	2.0	1.3E–02	2.3	3.1E–07	A DNase II homolog; required for DNA degradation during apoptosis, and degradation of dietary DNA
asm-3	14.1	2.3E–02	23.8	8.1E–40	Sphingomyelin phosphodiesterase 1 ortholog; determination of adult lifespan
asp-1^c^	2.3	2.7E–02	2.8	9.9E–09	Cathepsin D homolog (aspartic protease); transcribed in late embryo/early larvae intestine; not observed later
col-77	9.8	2.9E–02	32.0	6.5E–08	Encodes a cuticular collagen
C53B7.3^c^	2.0	3.2E–02	4.0	1.5E–10	Enriched in: germ line, intestine, PVD/OLL neurons
asp-3^c^	1.8	3.6E–02	2.3	2.9E–13	Encodes an aspartyl protease homolog, required for degenerative (necrotic-like) cell death in neurons
ZK896.5^c^	High^d^	3.9E–02	5.1	8.1E–03	Epoxide hydrolase 1 ortholog; enriched in: intestine, pharyngeal muscle

^a^Fold change in worms raised on complex microbiotas compared with worms raised on *E. coli*

^b^Based on WormBase version WS259

^c^Known expression in the intestine

^d^When expression in *E. coli* was undetected (0)

Among the genes upregulated by the exposure to diverse microbiotas were genes previously described to respond to environmental bacteria, specifically to a *Comamonas* isolate^33^ (11/25 genes, Supplementary Table 1). Although this is not surprising considering that SC1 included a *Comamonas* isolate, upregulation of these genes in worms raised in compost microcosms suggests similar interactions in natural-like environments, which is in agreement with previous reports of *Comamonas* species being part of the *C. elegans* gut microbiota^13,31^. Further in agreement with enrichment for immune genes, a significant overlap was observed between the microbiota-upregulated 25 genes and targets of central immune regulators, including the p38 pathway^34^, the DAF-16/FOXO transcription factor^35^, and TGFβ/ΒΜP signaling^36^ (Fig. 1a, Table 1 and Supplementary Table 1). However, how these regulators contributed to responses to, and interactions with complex microbiotas was not immediately obvious, as their targets appeared also among downregulated genes. An exception to this broad overlap was the specific enrichment of p38 targets among genes upregulated on SC1, potentially associated with the presence of a *Pseudomonas mendocina* isolate previously shown to prime p38-dependent immune responses^37^.

### TGFβ/BMP signaling is involved in shaping the gut microbiota

Since immunity emerged as a common denominator in worm responses to complex microbiotas, and immune regulators as potential players in shaping these responses, we tested the significance of disrupting such regulators for the composition of the worm gut microbiota. Mutants examined included *pmk-1(km25)* and *tir-1*(*qd4)*, disrupted for the p38 MAPK ortholog and its SARM adaptor, respectively^38^; *dbl-1(nk3)* mutants, lacking the TGFβ/BMP-like ligand DBL-1^39^; *daf-16(mu86)* mutants, disrupted for a FOXO transcription factor central for stress resistance, immunity, and longevity, along with mutants for one of its targets, the *ctl-2* catalase^35^; and *tol-1(nr2033)* mutants, disrupted for the sole toll receptor homolog in *C*. *elegans*^40^. As a control, we examined *tnt-3(ok1011)* mutants, which are defective in grinding ingested bacteria, allowing more intact bacteria into the intestine^41^. Beyond factors that operate in the intestinal niche, we examined mutants for *osm-6* and *ttx-3*, which are involved in feeding behavior, which might also affect microbiota structure^42^. All worm strains were raised on plates with the SC1 synthetic community, and their gut microbiota size (total bacterial load) and composition were evaluated in the first day of adulthood, using qPCR with eubacterial and taxa-specific primers, calibrated to known quantities of a full-length amplicon of the 16S ribosomal RNA gene (rDNA) from the respective taxa (see Methods). Measured taxa included *Enterobacteriaceae* and *Pseudomonadaceae*, which are the most abundant families in the *C. elegans* gut, as well as the less common *Bacillaceae*, which are nevertheless part of the worm core gut microbiota^13^.

The *tnt-3* control strain showed a significantly greater gut bacterial load than wild-type animals (Fig. 1b). This was expected, as it is impaired in the grinding of bacteria. In contrast, most tested mutants showed only small or insignificant changes in their total bacterial load. The exception was *dbl-1* mutants, which demonstrated a threefold increase. This was associated with increases in the abundance of *Enterobacteriaceae*, and more variably, of *Pseudomonadaceae* (Figs. 1c, d). The abundance of *Bacillaceae* family members in wild-type or mutant animals was variable to the extent that no clear differences could be discerned (Fig. 1e).

Although total bacterial load in *tnt-3* mutants was much larger than in wild-type animals, microbiota composition, represented by the relative abundance of measured taxa, did not significantly change, indicating proportional increases in different microbiota members (Fig. 1f). A similar trend in taxa relative abundance was observed in feeding behavior mutants, suggesting that at least in the context of the plate microbiota, food preference was not a significant factor shaping the gut microbiota. In contrast, mutants for stress and immunity regulators, which had the same total bacterial load as wild-type animals (excluding *dbl-1* mutants), showed significantly altered composition: *pmk-1*, *tir-1*, and *daf-16* mutants showed relative expansion in taxa (one or more) for which we did not have specific primers, i.e., *Comamonas*, *Aeromonas*, or others (bundled as “other”), on the expense of the taxa that in wild-type animals are the dominant ones, *Enterobacteriaceae* and *Pseudomonas* (Fig. 1f). *dbl-1* mutants, on the other hand, harboring an expanded gut microbiota, showed a significant increase in the relative abundance of *Enterobacteriaceae*. Both the increase in total bacterial load in *dbl-1* mutants, and the particular expansion of *Enterobacteriaceae* were confirmed with colony- forming unit (CFU) counts, evaluating the number of live bacteria in the intestine of wild-type and *dbl-1* animals (Fig. 1g).

### BMP/DBL-1 signaling specifically affects *Enterobacter* abundance

**Fig. 2:**
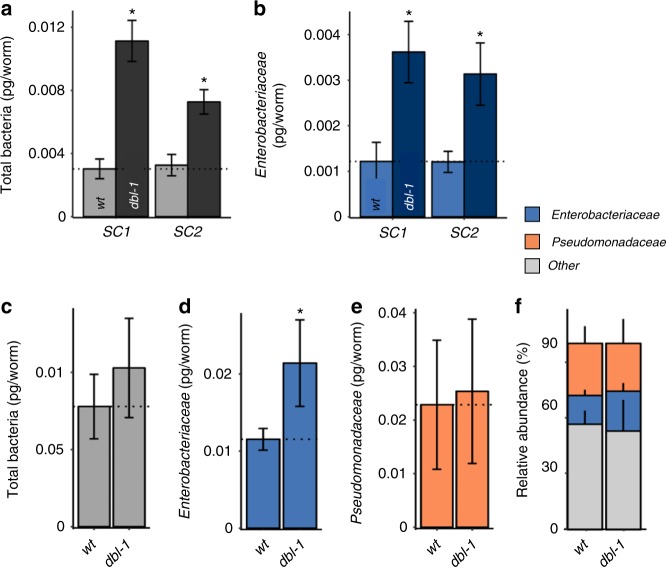
*Enterobacteriaceae* expansion in *dbl-1* mutants is reproduced in different environments. Total bacterial load (**a**), and abundance of *Enterobacteriaceae* (**b**) in the gut of wild-type (N2) or *dbl-1* worms raised on synthetic communities SC1 or SC2. Shown are averages ± SD of two independent experiments (*n* = 30 worms per group per experiment). **c**–**f** Total bacterial load (**c**), and abundance of *Enterobacteriaceae* (**d**) and *Pseudomonadaceae* (**e**), in worms raised in soil microcosms; **f** calculated relative abundance, as in Fig. 1. Averages ± SD of four independent populations per genotype (*n* = 100 worms per group). *p < 0.05 compared with wild-type animals, *t*-test

Focusing on the prominent effects of *dbl-1* disruption, we examined whether microbiota expansion in *dbl-1* mutants was a general trend in these mutants, or unique to the particular composition of SC1. To this end, we raised wild-type worms and *dbl-1* mutants on a different synthetic community (SC2), in which approximately two-thirds of SC1 strains were replaced with distinct isolates, while keeping the same genera represented (Supplementary Table 2). Both on SC1 and on SC2, *dbl-1* mutants showed a significantly greater bacterial load compared with wild-type worms ( *p* < 0.001, Fig. 2a), as well as a threefold more *Enterobacteriaceae* (Fig. 2b). Furthermore, when grown in composted soil microcosms, providing natural-like microbial diversity, *dbl-1* mutants showed only a modest increase in total bacterial load, but a significant twofold increase in the abundance of *Enterobacteriaceae* (Fig. 2c–f). Together, these observations support the notion that gut microbiota expansion in *dbl-1* mutants, and in particular an increase in the abundance of *Enterobacteriaceae*, represented a general feature of these mutants.

**Fig. 3:**
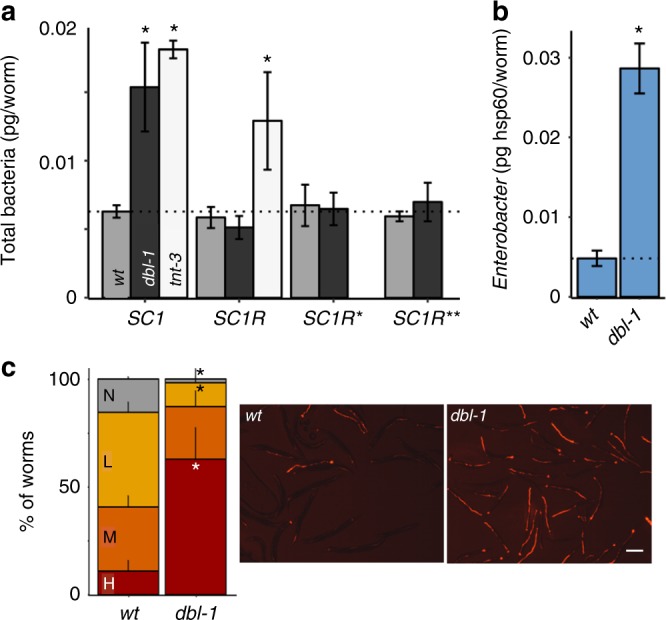
*dbl-1* disruption specifically affects *Enterobacter* species. **a** Microbiota size in worms of the designated strains raised on different versions of the SC1 synthetic community—without *Pseudomonas* and most of the *Enterobacteriaceae* isolates (SC1R), without most *Enterobacteriaceae* (SC1R*), or without *Enterobacter* isolates (SC1R**). Average ± SD of two independent populations (*n* = 30/experiment); *p < 0.05 compared with wild-type, *t*-test. **b** Measurements of the *Enterbacter* load (assessed with calibrated qPCR using *Enterobacter*-specific primers) in wild-type animals (N2) and *dbl-1* mutants raised on SC1. Average ± SD of two independent populations (*n* = 30 worms per population). *p < 0.05 compared with N2, *t-*test. **c** Representative images and quantification of worm colonization by tdTomato-expressing *Enterobacter cloacae CEent1*. H high, M moderate, L light, N none. Averages ± SD of two independent experiments (*n* = 62–180 worms per experiment). *p < 0.05, Tukey’s HSD test. Size bar, 0.3 mm

To determine whether any specificity could be discerned in the effects of *dbl-1* disruption on the gut microbiota, we created modified versions of SC1 with reduced diversity. The first modified community configuration, SC1R, excluded all *Pseudomonas* isolates, as well as most of the *Enterobacteriaceae* (Supplementary Table 2). While *dbl-1* mutants raised on SC1 have an expanded microbiota compared with wild-type animals, those raised on SC1R did not show this expansion (Fig. 3a), indicating that disruption of *dbl-1* had somewhat restricted effects, allowing a bloom of *Pseudomonas* and/or *Enterobacteriaceae*, but not of other members of the community. In contrast, *tnt-3* mutants raised on SC1R demonstrated an expanded microbiota similar to when raised on SC1, supporting the indiscriminate effects of defective grinding on microbiota expansion. A subtler modification of SC1, SC1R*, which excluded only *Enterobacteriaceae* species (not all), also abolished the increase in microbiota size in *dbl-1* mutants, further indicating specificity toward certain *Enterobacteriaceae* species. Finally, eliminating only *Enterobacter* isolates from the synthetic community (SC1R**), leaving-in several other *Enterobacteriaceae* species (e.g., *Escherichia sp., Buttiauxella sp*.), was sufficient to abolish microbiota expansion in *dbl-1* mutants, pointing at *Enterobacter* isolates as those affected by *dbl-1* disruption. Supporting this, re-examination of the microbiota in *dbl-1* mutants raised on SC1, using primers specific for the *Enterobacter hsp60* gene, revealed an increase in the *Enterobacter* load in *dbl-1* mutants that could account for the entire increase in bacterial load observed in these animals (Fig. 3b, as compared with Fig. 3a).

The observed increase in *Enterobacter* abundance in *dbl-1* mutants could be due to direct effects of host TGFβ/BMP signaling on *Enterobacter* colonization and/or proliferation, or caused indirectly by changes in microbial interactions affecting the balance between *Enterobacter* and its competitors. To examine which of the two alternatives is more likely, we raised worms on a tdTomato-expressing derivative of one of the SC1 isolates, a previously characterized *Enterobacter cloacae* commensal of *C. elegans*, *CEN2ent1* (shortened here to *CEent1*)^18^. Fluorescent microscopy of adult *dbl-1* and wild-type animals demonstrated a significant increase in the abundance of these bacteria in *dbl-1* mutants compared with wild-type animals (Fig. 3c). As no other bacteria are present to affect *CEent1* abundance, these results indicate that *dbl-1* disruption directly affected *Enterobacter* abundance.

### *Enterobacter* bloom is not due to mutants’ impaired development

**Fig. 4:**
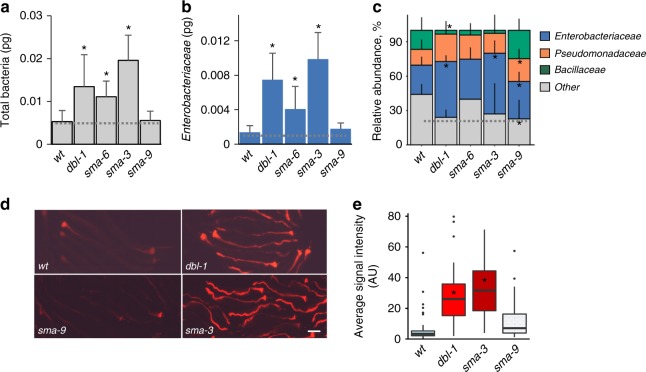
Disruption of TGFβ/BMP signaling alters gut microbiota size and composition independent of effects on body size. **a**, **b** Bacterial load in wild-type worms and TGFβ mutants—*dbl-1(nk3), sma-6(wk7), sma-3(e491)*, and *sma-9(wk55)*, raised on the SC1 community, showing abundance of all *Eubacteria* (**a**), or *Enterobacteriaceae* (**b**). Average ± SD of two independent experiments. Measurements were performed on a pool of *n* = 30 worms per experiment. **c** Relative abundance of all measured taxa (including also *Pseudomonadaceae* and *Bacillaceae*), calculated based on the absolute quantities, as in Fig. 1; light gray bar represents relative abundance of all ‘other’ bacterial groups not directly measured. *p < 0.05 (analysis of variance (ANOVA)) compared with wild-type animals. **d**, **e** Representative images (**d**) and quantification (**e**) of colonization of TGFβ/BMP mutants by tdTomato-expressing *CEent1*. Box plots present medians (center line), first and third quartiles (bottom and top of box, respectively), minimum and maximum values (whiskers), and outliers (dots, defined as >1.5 times above/below the interquartile range). *p < 10E–13 (generalized linear model) compared with wild-type animals; *N*(*wt)* = 47, *N*(*dbl-1*) = 42, *N*(*sma-3*) = 66, *N*(*sma-9*) = 89. Size bar, 0.1 mm

TGFβ/BMP signaling involves ligand binding and activation of heterodimer receptors, downstream SMAD transcriptional regulators (Sma and Mothers against decapentaplegic homologs), and co-activators^43^. In *C. elegans*, this pathway contributes to immunity, but more visibly to development, and all known mutants have small body size. Whereas the smaller *dbl-1* mutants showed a greater bacterial load than wild-type animals, we nevertheless wished to ascertain that this bloom was not caused (perhaps indirectly) by altered development. Disruption of the TGFβ type I receptor gene, *sma-6*, or the R-SMAD (Receptor-regulated SMAD) gene, *sma-3*, both led to an *Enterobacteriaceae* expansion, either slightly smaller than in *dbl-1* mutants (*sma-6*) or larger (*sma-3*) (Fig. 4a–c). In contrast, mutants for the *sma-9* co-activator, which are as small as *dbl-1* mutants^44^, showed no bacterial expansion whatsoever (Fig. 4a, b). Fluorescent imaging, using the tdTomato-expressing *CEent1* to follow *Enterobacter* colonization, corroborated these results (Fig. 4d, e), further diminishing the likelihood that *Enterobacter* bloom in TGFβ/BMP mutants was due to altered development in mutants. This in turn leaves impaired immunity as the more likely cause for the *Enterobacter* bloom. In line with this, while *sma-6*, *sma-3*, and *dbl-1* are central immune regulators, *sma-9* was reported to be redundant for immune gene induction^45^.

### TGFβ/BMP-exerted control acts primarily in the anterior gut

**Fig. 5:**
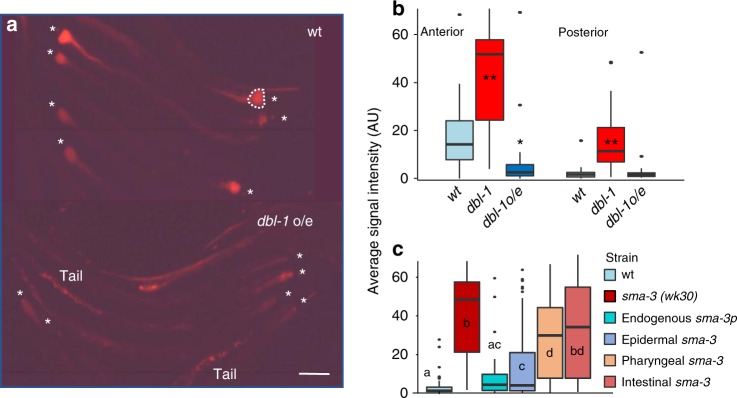
TGFβ/BMP signaling controls *Enterobacter* colonization in the anterior intestine through multi-tissue contributions. **a** Representative images of wild-type and *dbl-1* overexpressing (o/e) worms raised to adulthood on tdTomato-expressing *CEent1*. Asterisks mark the posterior pharynx, size bar, 0.1 mm. **b** Box plots (described under Fig. 4) quantifying fluorescent signal (normalized to area) in images as in **a**, either in the anterior gut (dashed line in **a**), or posterior to that. *n* = 38 (wt), 50 (*dbl-1*), 28 (dbl-1 o/e); **p < 0.0001, *p < 0.05, generalized linear model. **c** Similar quantification in the anterior gut of wild-type worms, or *sma-3* mutants, as well as in *sma-3* derivatives with transgenic tissue-specific *sma-3* expression. Statistically distinct groups (*p* < 0.05) are marked with different letters (analysis of variance (ANOVA) followed by a Tukey’s post hoc test; *n* = 71(*wt*), 60 (*sma-3 mutants*), 48 (endogenous sma-3 promoter), 86 (epidermal *sma-3*), 40 (pharyngeal *sma-3*), 33 (intestinal *sma-3*), all hermaphrodites)

To examine the opposite case of *dbl-1* disruption, we employed a strain overexpressing *dbl-1* from an integrated genomic fragment (*dbl-1 o/e*)^39^. As observed in animals raised to adulthood on *tdTomato-CEent1, Enterobacter* load in *dbl-1 o/e* animals was overall comparable to that in wild-type animals (Fig. 5a, b). However, the distribution of bacteria in the gut was very different: although wild-type animals showed prominent accumulation of *Enterobacter* in the anterior gut, this was mostly missing in *dbl-1 o/e* animals (Fig. 5a, b). This suggested that TGFβ/BMP signaling exerted its control over *Enterobacter* colonization/proliferation mainly in the anterior gut.

DBL-1 is expressed primarily in neurons, but its receptors and downstream mediators are expressed in the epidermis, pharynx and in the intestine^43^. To gain insight into where TGFβ/BMP signaling might function to delimit *Enterobacter* accumulation, we took advantage of *sma-3(wk30)* mutants, which are heavily colonized with *Enterobacter*, and a panel of transgenic strains employing tissue-specific promoters to rescue *sma-3* expression in different tissues^46^. Expression of *sma-3* from its endogenous promoter effectively restored accumulation of *tdTomato-CEent1* to wild-type levels (Fig. 5c). Expression of *sma-3* from the epidermal *dpy-7* promoter also delimited *Enterobacter* accumulation, although not quite to wild-type levels, and expression from the pharyngeal *myo-2* promoter showed *Enterobacter* accumulation to levels intermediate between those of wild-type and those in *sma-3* animals. In contrast, worms with intestinal *sma-3* expression (relying on the *vha-6* promoter) showed *Enterobacter* accumulation that was not significantly different from that seen in *sma-3* mutants. Similar trends were observed in measurements performed on the anterior gut or on its posterior parts (Supplementary Fig. 3). These results suggest multi-tissue contributions of TGFβ/BMP signaling to controlling intestinal *Enterobacter*, with the epidermis providing the more dominant input, whereas local TGFβ/BMP signaling in the intestine appearing to be mostly redundant.

### TGFβ/BMP disruption turns an *Enterobacter* commensal to pathogenic

**Fig. 6:**
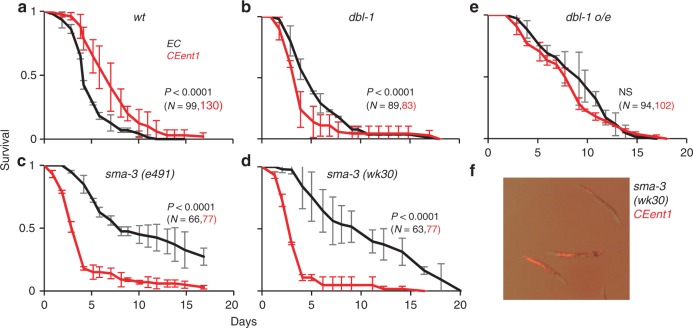
Normally beneficial *Enterobacter* commensal is detrimental in *dbl-1* mutants. **a**–**e** Fraction survival of worms of the designated strains on the pathogen *E. faecalis*, following growth (until larval stage L4) on control *E. coli (EC)* or *CEent1*. Shown are averages ± SDs for representative experiments performed in duplicate or triplicate. *P*-values were obtained using log-rank test. **f** An overlay of visible light and fluorescent images demonstrating colonization of *sma-3* mutants with tdTomato-expressing *CEent1* 24 h after shift from *CEent1* to *E. faecalis* (a representative image from one experiment of two showing similar results)*;* size bar, 0.2 mm

*CEent1* was previously shown to be beneficial for *C. elegans*, accelerating development compared with worms raised on the standard *E. coli* food, and enhancing resistance to the pathogen *Enterococcus faecalis*^18^ (Fig. 6a). A 4-h exposure of worms to the commensal late in development (at the L4 stage) was sufficient to increase resistance to subsequent infection (Supplementary Table 3). However, *dbl-1* mutants developing on *CEent1* (or exposed to it late in development) no longer showed enhanced pathogen resistance and instead were significantly more susceptible (Fig. 6b and Supplementary Table 3). *sma-3* mutants showed an even greater susceptibility (Fig. 6c, d). Furthermore, *sma-3* mutants raised on tdTomato-*CEent1* and shifted late in development to pathogen were still colonized 24 h after the shift (9 out of 24 examined) (Fig. 6f), and even 48 h after the shift, at which point colonization was further observed in three out of five cadavers. In contrast, wild-type animals showed no *CEent1* colonization persisting after the shift. These results indicate that impaired TGFβ/BMP signaling results in a more persistent *Enterobacter* colonization, turning the normally beneficial commensal to pathogenic. Interestingly, *dbl-1* overexpressing worms, which were more resistant to *E. faecalis* to begin with, showed no significant increase in their resistance when initially exposed to *CEent1* instead of *E. coli* (Fig. 6e).

## Discussion

With the microbiome linked to many aspects of host health, understanding the interactions between host and its microbes is imperative for the development of therapeutic strategies aiming to alter the microbiota. An important aspect of this is understanding how host genes determine abundance and function of gut commensals. This understanding has been slow to emerge, to some extent due to inter-individual variation in microbiota composition in vertebrate models, which masks gene effects. Here, we demonstrate the utility of *C. elegans* as a model organism facilitating the identification and characterization of such gene effects, including the distinction between effects on microbiota size (total microbial load) and microbiota composition. Using this model, and screening candidate regulators, we identified a role for TGFβ/BMP immune signaling in controlling the abundance of members of the genus *Enterobacter*, common inhabitants of the worm gut, which affect host development and immunity. TGFβ/BMP-exerted control was focused on restricting *Enterobacter* accumulation in the anterior gut, but full effects depended on contributions from several tissues. Disruption of TGFβ/BMP signaling resulted in an *Enterobacter* bloom, and turned an otherwise useful commensal to pathogenic. Gut dysbiosis is typically considered a condition that enables invasion and proliferation of environmental opportunistic pathogens. Our results demonstrate that given impairment in host immunity and ensuing dysbiosis, pathogenicity can emerge directly from otherwise beneficial members of the gut community.

Current understanding of how host genes and processes shape microbiota composition highlights the importance of mucosal structure and immunity. Genes identified as affecting abundance of gut bacteria include antimicrobial peptides, pattern recognition receptors, cytokines, and genes affecting IgA antibody production^47–49^, as well as enzymes glycosylating barrier mucins, which provide sugars harvested by gut bacteria^50,51^. Our RNAseq analysis indicated a similar trend in *C. elegans*, as worms interacting with a diverse microbiota had elevated expression of genes, mostly intestinal, which are associated with host immunity. Some of these genes encode hydrolytic enzymes, which may serve immune functions, but could also be relevant for releasing nutrients from susceptible ingested bacteria for the benefit of resistant gut commensals. Mutant analysis supported the importance of host immunity in shaping the gut microbiota. Since many of the identified overexpressed genes are members of multi-gene families, implying potential functional redundancy, we focused instead on central upstream immune regulators. The contribution of immune regulators to microbiota composition was conspicuous, and in the case of *dbl-1* mutants was recapitulated with different synthetic communities, as well as (with regards to the main feature of *Enterobacteriaceae* expansion) in worms raised in a natural-like compost microcosm. In contrast, disruption of *daf-16*, which encodes a general stress resistance regulator and contributes also to immunity, had no effect on microbiota composition^52^. Feeding behavior may also be thought to affect microbiota composition. However, disruption of *osm-6* and *ttx-3*, did not show any effect. This cannot completely rule out involvement of food sensing and feeding behavior in determining microbiota composition, as the synthetic microbiota used is solely made of gut isolates (i.e., commensals) and is thoroughly mixed; under more natural conditions regulators of feeding behavior may contribute by identifying pockets of desirable bacteria in a non-homogeneous environment. Nevertheless, the finding that impaired grinding in *tnt-3* mutants affected microbiota size but not composition supports an important role for post-feeding mechanisms, likely those defining the intestinal niche, in shaping microbiota composition.

TGFβ/BMP signaling regulates diverse processes in *C. elegans*, including growth, male tail development, and immunity^43^. Our results point at its roles in immune regulation as those affecting gut bacterial load. To date, not much is known about how TGFβ/BMP signaling regulates intestinal immunity^53,54^. Our results indicate that in controlling commensal colonization it relied on multi-tissue contributions, primarily from the epidermis, but also from the pharynx. Contributions of TGFβ/BMP signaling in the intestinal niche itself were marginal at best (although it seemed to affect *Enterobacter* accumulation in the posterior gut more than it did in the anterior, see Supplementary Fig. 3). This suggests that TGFβ/BMP signaling may affect the *Enterobacter* niche indirectly, potentially involving an additional signaling pathway that targets the gut. This mode of regulation may also apply to TGFβ/BMP involvement in resistance to intestinal pathogens. At the same time, results from experiments with *dbl-1 o/e* worms suggest that the *Enterobacter* niche in question is the anterior gut, as overexpression of the ligand and the presumed over-activation of the pathway prevented *Enterobacter* accumulation particularly in that region. It is further possible that colonization of this region is essential for the protective effects of *Enterobacter*, explaining the inability of *CEent1* to enhance infection resistance in *dbl-1 o/e* animals.

The role of immunity in shaping the gut microbiota may be thought to be nonspecific, especially if considering the reliance of *C. elegans* solely on innate immunity (as all invertebrates). If so, disruption of any immune pathway will cause a relatively indiscriminate proliferation of gut commensals. However, this was not the case, as bacterial proliferation was selective and specific to the disrupted pathway: disruption of TGFβ/BMP signaling led to blooming only of *Enterobacter* species, not affecting other members of the microbiota, including other isolates of the *Enterobacteriaceae* family; and disruption of p38 signaling did not affect the abundance of major examined taxa (*Pseudomonadaceae* and *Enterobacteriaceae*), and only caused an increase in relative abundance of other taxa, yet to be defined. It might be speculated that such specificity depends on the profile of immune effectors regulated by each of the pathways, and differential susceptibility of different gut microbes to components of these profiles.

TGFβ signaling is highly conserved, regulating development and immunity also in vertebrates^55^. Whereas TGFβ/BMP signaling was shown to be associated with immune responses both in *Drosophila* and in vertebrates^56,57^, it is the role of TGFβ signaling in vertebrate T-cell differentiation and mucosal homeostasis that is better known^58^. Of particular relevance is the role of TGFβ signaling in production of IgA antibodies, which delimit microbiota proliferation in the gut^59^. Early results showed that TGFβ1 deficiency correlated with ulcerative colitis-associated colon cancer, and studies in mice suggested that colitis and cancer might depend on gut microbes^60^. More recently, it has been shown that deficiency in TGFβ signaling in intestinal dendritic cells led to changes in epithelial structure, *Enterobacteriaceae*-driven dysbiosis, and colitis^61^. Together, these reports describe a central role for TGFβ signaling in controlling gut microbial proliferation. Our results in *C. elegans* suggest that this role is conserved, even without specialized immune cells or antibodies, and further suggest particular importance, with ancient origins, for TGFβ signaling in controlling gut commensals of the *Enterobacteriaceae* family.

## Methods

### *C. elegans* strains and maintenance

Strains used in this study included N2 wild-type worms, the mutant strains *pmk-1(km25), tir-1(qd4), tol-1(nr2033), dbl-1(nk3), sma-3(e491), sma-6(wk7), sma-9(wk55), daf-16(mu86), ctl-2(ok1137), osm-6(p811), ttx-3(ot22)*, and *tnt-3(ok1011)*, and the *dbl-1* overexpressing strain BW1940 (which harbors several copies of the genomic *dbl-1* locus), all acquired from the *Caenorhabditis* Genetic Center. In addition, strains with tissue-specific expression, gratefully received from Meng Wang’s lab, included *sma-3(wk30);him-5(e1490);qcEx24[sma-3p::GFP::sma-3* *+* *rol-6]*, *sma-3(wk30); him-5(e1490);qcEx52[myo-2p::GFP::sma-3* *+* *rol-6]*, *sma-3(wk30);him-5(e1490);qcEx53[vha-6p::GFP::sma-3* *+* *rol-6]*, and *sma-3(wk30);him-5(e1490);qcEx5[dpy-7p::GFP::sma-3* *+* *rol-6]. sma-3(wk30);him-5(e1490)* were obtained by picking non-rollers, who lost the extrachromosomal transgene. All strains were cultured on nematode growth media (NGM) at 25 °C.

### Worm growth in soil and harvesting

Fresh local soil was supplemented with chopped over-ripe apples. The soil produce mixture was allowed to decompose for 2 weeks in the lab, cleared of garden variety worms by autoclaving, and re-inoculated with a microbial extract from the original batch of unautoclaved soil 24 h prior to addition of worms (extracts were the supernatants (1800 rpm) from soil resuspended and vortexed in M9 salt solution)^13^. Initially germ-free L1 larvae, obtained by bleaching of gravid worms to release eggs, and hatching them on NGM plates without food, were transferred to soil and grown at 25 °C for 3 days. One batch of prepared soil was split into separate 20 mL glass beakers (5 g per vial), and independent worm populations were raised in each (four replicates per genotype). Approximately 100–200 gravid worms were harvested from each population using a Baermann funnel lined with two layers of tissue paper, washed extensively (6 × ), and surface sterilized on plates containing 100 μg/ml gentamicin prior to DNA extraction, using the MO BIO PowerSoil DNA isolation kit (#12888)^13^.

### Synthetic community and worm growth

Bacteria were previously isolated from worms grown in soil, and identified by 16S sequencing or multi-locus sequencing^13,18,37^. *Enterobacteriaceae* isolates were cultured on an *Enterobacteriaceae-*selective medium (Violet Red Bile Glucose, VRBG), whereas all others were cultured on Lysogeny Broth (LB) agar plates. A total of 30 isolates were selected for the synthetic community, representing most core taxa of the previously characterized *C. elegans* gut microbiota taxa^13^ (Supplementary Table 2). Several configurations of this community were used, including SC1 and SC2, which include the same genera, but differ in the specific strains used; SC1R resembles SC1, but lacks *Enterobacteriaceae* and *Pseudomonas* isolates; SC1R* lacks most of the *Enterobacteriaceae* isolates; and SC1R** contains all the SC1 isolates, except for the *Enterobacter* isolates. For each microbiota, isolates were grown in 1 mL of LB broth at 37 °C overnight with shaking. Saturated cultures were combined in equal proportions, concentrated 10 × , and seeded onto NGM plates approximately 30 min before the addition of eggs, obtained from bleaching gravid worms.

For each worm strain, 2–3 independent populations of worms, synchronized at L1, were grown on the synthetic microbiotas at 25 °C for 3 days. Adult worms were washed, surface sterilized, and used for DNA extraction, as described for worms raised on soil.

### DNA extraction

For each plate, 30 washed worms were transferred to a lysis buffer of a constant volume (per 30 worms: 6 μl of 10X PCR buffer (Invitrogen #11304011), 3.6 μl of Proteinase K (Fisher Scientific #EO0491), and 50.4 μl of PCR-grade water), and were lysed at 60 °C for 1 h, followed by a 15-min incubation at 95 °C to inactivate the proteinase K. Samples were stored at –20 °C until use. To rule out measurement biases introduced by DNA extraction method, DNA was extracted from synthetic communities mixed in different ratios either using the proteinase K protocol, or the MO BIO PowerSoil DNA isolation kit (#12888). Comparisons (demonstrating similar measurements of microbiota composition) are shown in Supplementary Fig. 4.

### RNAseq library preparation and analysis

Synchronized worm populations, initiated with eggs obtained from bleached gravid worms, were grown on either NGM plates seeded with *E. coli*, NGM plates seeded with the synthetic community, in autoclaved soil supplemented with *E. coli*, or in composted soil, as described above (three independent populations per condition). RNA was extracted from gravid worms using a modified CTAB protocol with Aluminum Ammonium Sulfate and PEG precipitation^62^, followed by purification with the Qiagen AllPrep kit (#80204) to separate RNA from DNA. Sequencing libraries were prepared from total RNA using the Illumina TruSeq RNA Library Kit v2 (RS-122-2001), which synthesizes complementary DNA (cDNA) from mRNA fragments using random primers, and provides 24 indexed adapters for multiplex sequencing. Paired-end sequencing was performed on Illumina HiSeq4000, generating 100 base-pair reads, from which adapter sequences, as well as low-quality reads, were removed prior to analysis, providing 30,270,407 reads/sample on average. Both forward and reverse sequences were used for analysis, employing Kallisto to identify and quantify transcripts, and Sleuth to identify differentially expressed genes, using a false discovery rate-corrected *p-*value (*q-*value) cutoff of 0.05, as previously described^63^.

GoTermFinder was used to identify enrichment among identified genes for representatives of annotated processes or functions. Enrichment for gene targets of various regulators was calculated using the hypergeometric test.

### RNA extraction and qRT-PCR

RNA was extracted from 100 to 200 worms per group using Trizol (Invitrogen). Genomic DNA contamination was removed with Turbo DNase (QIAGEN), cDNA was synthesized using iScriptTM (Bio-Rad), and quantitative real-time PCR was carried out using Bio-Rad’s SsoAdvanced Universal SYBR Green Supermix, and on a StepOnePlus system (Applied Bio). Ct values were normalized to the respective actin values for each sample, and are presented as fold change over wild-type worm expression value. See Supplementary Table 4 for primer sequences.

### Quantifying bacterial load (microbiota size) and microbe abundance

Quantitative PCR (qPCR) was used to estimate taxa abundance by quantifying their respective DNA (in pg). Eubacterial 16S rDNA was quantified using the conserved primers 806f and 895r (Supplementary Table 4), and taxa-specific primers were used to quantify *Enterobacteriaceae* 16S (Ent_MB_F and R), *Pseudomonadaceae* 16S (Pse435F, Pse686R), and *Bacilli* 16S (BacilliF, BacilliR) in DNA extracts from worm samples obtained either using standard proteinase K based lysis, or PowerSoil, as described above. In addition, taxa-specific primers targeting the Hsp60 gene were used to quantify *Enterobacter cloacae* (Ent-Hsp60f, Ent-Hsp60r). Cycling parameters: 95 °C for 5 min; 45 cycles of 95 °C for 15 s, 60 °C for 30 s, 72 °C for 15 s [30 s for *Bacilli*]; 72 °C for 5 min. Specificity of each primer pair used was confirmed by PCR on each individual member of the synthetic communities, further ensuring no amplification of *C. elegans* mitochondrial DNA, which can be amplified with some 16S primers. All measurements were performed in duplicate with 1 μl of DNA extract as template, prepared from 30 worms in 60 μl of lysis buffer (for worms grown on synthetic microbiotas) or from 100 worms in 30 μl (DNA extraction from worms grown on soil), to enable comparison of values between worm populations. DNA quantities were estimated using standard curves created for each taxa from qPCR measurements of 10-fold serial dilutions of an appropriate full-length 16S bacterial rDNA in known concentrations, ranging from 25 ng to 0.2 pg/μl (amplified using the same primers as described above); *E. coli* 16S was used for calibrating both eubacterial and *Enterobacteriaceae* measurements, *Pseudomonas mendocina* 16S for *Pseuomonadaceae* measurements, *Bacillus subtilis* 16S for *Bacilli* measurements, and a 500-bp region of the Hsp60 gene from *Enterobacter cloacae* for *Enterobacter* Hsp60 measurements. Relative abundance was estimated by dividing the quantity of DNA from each bacterial group by the quantity measured with the universal primers. The ability to estimate relative abundance of bacterial families in a gut community was validated by measuring abundance of members of the different families in plated synthetic communities prepared with different ratios of members of these families. Measured relative abundances reflected the expected mix ratios and were unaffected by the method used for DNA extraction (Supplementary Fig. 4).

### CFU counts

Following harvesting and washing, worms were ground (10 worms in 250 μl of M9), releasing live bacteria, which were serially diluted. Aliquots were plated onto either LB or VRBG plates, which were incubated at 37 °C overnight before counting.

### Fluorescently tagged Enterobacter cloacae

A plasmid that constitutively expresses tdTomato from the *Enterobacter* cysB promotor (fragment 132a)^64^, with kanamycin resistance as a selection marker, was constructed at the UC Berkeley MacroLab. The plasmid was introduced into a *C. elegans* commensal, *CEN2ent1*, which is naturally ampicillin resistant^18^, using a triparental mating approach^65^. Briefly, the tdTomato plasmid was transformed into competent DH5ɑ *E. coli* cells using a standard heat shock protocol. *CEN2ent1*, DH5ɑ-tdTomato, and the DH5ɑ *E. coli* helper strain, which harbors the pRK2073 F conjugative plasmid with streptomycin resistance, were separately streaked out onto LB plates with either ampicillin, kanamycin, or streptomycin, respectively, and incubated at 37 °C overnight. Cells were scraped off from each plate, mixed together on a new LB plate, and incubated overnight at 37 °C. Colonies were streaked onto a new LB plate with ampicillin and kanamycin to select for *CEN2ent1*-tdTomato cells. The isolate was confirmed to not have the conjugative plasmid by testing for streptomycin sensitivity and was confirmed to be *CEN2ent1* by sequencing of the full-length 16S gene.

### Imaging

*CEN2ent1*-tdTomato was grown overnight in LB broth with 100 μg/ml kanamycin. Cells were concentrated 10× and seeded onto NGM with 100 μg/ml kanamycin before eggs, obtained by bleaching, were added. After 3 days at 25 °C, worms were washed off the plates, and washed three times more with M9 buffer to remove external bacteria. Fluorescent images were captured using a Leica MZ16F equipped with a QImaging MicroPublisher 5.0 camera. Worm colonization was scored based on degree of colonization: “high colonization”, for colonization throughout the intestine; “moderate colonization”, for colonization in no more than half the length of the intestine; “light colonization”, for faint colonization, or “no colonization”. Alternatively, colonization was quantified using ImageJ, by drawing a line selecting the gut area for each worm and quantifying background-subtracted average intensity in this area. When relevant, signal intensity in the anterior and posterior parts of the intestine were quantified separately.

### Survival analyses

Worms were exposed to *CEent1* (or to control *E. coli* OP50) from the egg stage till larval L4 stage, or shifted to such plates as L4 larvae, for a 4-h exposure. Subsequently, worms were transferred to plates (Brain Heart Infusion agar, 20 µg/mL gentamicin) pre-plated with the pathogen *Enterococcus faecalis* strain V583^66^. Dead worms were counted to assess infection resistance. All experiments were carried out at 25 °C.

### Statistical analyses

Comparisons of worm fecundity, qRT-PCR measurements, and CFU counts were evaluated using two-sided Student’s *t*-test. qPCR measurements of microbiota size/total bacterial load and taxa-specific bacterial abundance were evaluated using a one-way analysis of variance (ANOVA) for repeated measures, to account for within-experiment replication (lme function from the *nlme* R package), with host genotype as a fixed effect, and within-experiment replication as a random effect^67^. Differences in worm colonization by *CEent1::GFP* were evaluated using ANOVA (*aov* function in R) followed by Tukey’s Honest Significant Difference (HSD) post hoc test when all comparisons were relevant, or linear model when one condition could serve as a reference for comparisons. Kaplan–Meier analysis was used to evaluate survival experiments, followed by a two-sided log-rank test, or a two-sided Wilcoxon test, which assigns greater weight to early time points, representing the bulk of the population.

### Reporting summary

Further information on experimental design is available in the Nature Research Reporting Summary linked to this article.

## Supplementary information

Supplementary Information

Description of Additional Supplementary Files

Supplementary Data 1

Supplementary Data 2

Reporting Summary

## Data Availability

All relevant data are available from the corresponding author. RNAseq raw data are available at: https://www.ncbi.nlm.nih.gov/geo/query/acc.cgi?acc=GSE97934.
