# Supplementary material for: TGFβ/BMP immune signaling affects abundance and function of C. elegans gut commensals
Source: Nat Commun. 2019 Feb 5;10:604. doi: 10.1038/s41467-019-08379-8 (PMC6363772; doi:10.1038/s41467-019-08379-8)
Supplement: Supplementary file 1 — Supplementary Information [file 41467_2019_8379_MOESM1_ESM.pdf]

TGFβ/BMP immune signaling affects abundance and function of *C. elegans* gut commensals

Berg et al.

Supplementary information

Including:
Supplementary Tables 1-4
Supplementary Figures 1-5
Supplementary references

Supplementary Table 1. Microbiota-dependent differentially expressed genes: overlaps with microbe responses and immune targets.

Gene list intersections #genes ([a]p-value)	[b]*Comamonas* ↑ (1187 genes)	[b]*Comamonas* ↓ (414)	[c]*E. coli* HT115 ↑ (31)	[d]*Pseudomonas* ↑ (197)	[e]TGFβ (217)	[f] PMK-1 (88)	[f] SEK-1 (101)	[g] DAF-16 (262)
**SC1 ↑ (127 genes)**	33 (2.5E-13)	2 (NS)	11 (3.3E-17)	21 (5E-20)	9 (9E-6)	12 (3E-13)	18 (2E-21)	9 (4E-5)
**SC1 ↓ (163)**	15 (0.03)	51 (5E-46)	0 (NS)	0 (NS)	7 (0.001)	2 (NS)	6 (0.0001)	10 (6E-6)
**Soil ↑(1269)**	138 (1E-12)	54 (2E-7)	5 (0.03)	22 (0.003)	17 (NS)	3 (NS)	8 (NS)	53 (3E-14)
**Soil ↓ (1815)**	105 (0.04)	60 (9E-5)	8 (0.004)	29 (0.003)	23 (NS)	14 (0.01)	21 (0.0002)	26 (NS)
**SC1↑ AND Soil↑ (25)**	11 (6E-8)	0	3 (8E-6)	5 (4E-6)	3 (0.002)	1(NS)	4 (7E-6)	4(3E-4)

[a] Hypergeometric test.

[b] In gravid worms

[c] In young adults

[d] Shapira M. *et al*. 2006[1]

[e] Roberts A.F. *et al*. 2010[2]

[f] PMK-1 is the *C. elegans* p38 ortholog; SEK-1 is the MAPK kinase that activates it[3]

[g] Murphy C.T. *et al.* 2003[4]

Supplementary Table 2. Gut isolates of SC1 (a) and SC2 (b).

a SC1 isolates

ID	[a,b] Genus/species	Family	SC1R	SC1R*	SC1R**
WG-2.4	*Arthrobacter*	*Micrococcaceae*	+	+	+
WG-2.5	*Microbacterium*	*Microbacteriaceae*	+	+	+
cre-6.1	*Aeromonas*	*Aeromonadaceae*	+	+	+
cre-5.3	*Comamonas*	*Comamonadaceae*	+	+	+
cre-1.1	*Comamonas*	*Comamonadaceae*	+	+	+
cre-1.2	*Shewanella*	*Shewanellaceae*	+	+	+
YA-3L	*Stenotrophomonas*	*Xanthomonadaceae*	+	+	+
L3-3L	*Sphingobacterium*	*Sphingobacteriaceae*	+	+	+
19.3.8	*Buttiauxella*	*Enterobacteriaceae*	+	+	+
19.3.3	*Rahnella*	*Enterobacteriaceae*	+	+	+
WG-1.2	*Achromobacter/Bordatella*	*Alcaligenaceae*	+	+	+
WG-2.1/ CEN2ent1	*Enterobacter cloacae*[5]	*Enterobacteriaceae*	-	-	-
cre-5.2	*Enterobacter* (soil strain)	*Enterobacteriaceae*	-	-	-
19.3.1	*Enterobacter*	*Enterobacteriaceae*	-	-	-
oak-7.1	*Escherichia*	*Enterobacteriaceae*	-	-	+
oak-5.2	*Lelliottia*	*Enterobacteriaceae*	-	-	+
euc-1	*Escherichia*	*Enterobacteriaceae*	-	+	+
WG-2.2	*Pseudomonas*	*Pseudomonadaceae*	-	+	+
2.2/ CEN2mendo	*Pseudomonas mendocina*[6]	*Pseudomonadaceae*	-	+	+
19.1.9	*Lelliottia*	*Enterobacteriaceae*	-	+	+
19.3.12	*Pseudomonas*	*Pseudomonadaceae*	-	+	+
2.4	*Bacillus*	*Bacillaceae*	+	+	+
3.2	*Bacillus*	*Bacillaceae*	+	+	+
3.1	*Bacillus*	*Bacillaceae*	+	+	+
2.1	*Bacillus*	*Bacillaceae*	+	+	+
14.1	*Bacillus*	*Bacillaceae*	+	+	+
3.7	*Paenibacillus*	*Paenibacillaceae*	+	+	+
10.1	*Lysinibacillus*	*Bacillaceae*	+	+	+
10.3	*Lysinibacillus*	*Bacillaceae*	+	+	+
19.1.7	*Lysinibacillus*	*Bacillaceae*	+	+	+

[a]Isolates were identified via BLAST of 16S sequences, rounding-up species-level ambiguities to the genus level.

[b]Isolates that are members of core microbiota families[7] are marked in blue; of nine core families, six are represented in SC1, although not necessarily in the same proportions as in worms raised in compost microcosms (e.g. *Pseudomonadaceae* are under-represented, *Bacillaceae* are over-represented). Isolates whose presence was typical of some microbiotas, and missing in others (two distinct microbiota types were identified in [7]) are shown in orange.

b SC2 isolates

[a]**ID**	[a]**Genus/species**	**Family**
WG-2.5	*Microbacterium*	*Microbacteriaceae*
cre-4.1	*Aeromonas*	*Aeromonadaceae*
cre-5.3	*Comamonas*	*Comamonadaceae*
cre-1.1	*Comamonas*	*Comamonadaceae*
cre-1.2	*Shewanella*	*Shewanellaceae*
YA-3L	*Stenotrophomonas*	*Xanthomonadaceae*
L3-3L	*Sphingobacterium*	*Sphingobacteriaceae*
3.4	*Arthrobacter*	*Micrococcaceae*
19.3.2 (isolated at 25ºC)	*Rahnella*	*Enterobacteriaceae*
19.3.8	*Buttiauxella*	*Enterobacteriaceae*
WG-1.2	*Paracoccus*	*Rhodobacteraceae*
YA-1v	*Enterobacter*	*Enterobacteriaceae*
19.3.2 (isolated at 37ºC)	*Enterobacter*	*Enterobacteriaceae*
WG-1.7/CEN2ent3	*Enterobacter cloacae*	*Enterobacteriaceae*
cre-5.2	*Enterobacter (soil strain)*	*Enterobacteriaceae*
oak-7.2	*Escherichia*	*Enterobacteriaceae*
euc-1	*Escherichia*	*Enterobacteriaceae*
WG-2.2	*Pseudomonas*	*Pseudomonadaceae*
2.3	*Pseudomonas mendocina*	*Pseudomonadaceae*
19.3.5	*Citrobacter*	*Enterobacteriaceae*
19.1.8	*Pseudomonas*	*Pseudomonadaceae*
WG-1.8	*Pantoea*	*Enterobacteriaceae*
2.5	*Bacillus*	*Bacillaceae*
3.3	*Bacillus*	*Bacillaceae*
19.3.9	*Citrobacter*	*Enterobacteriaceae*
2.1	*Bacillus*	*Bacillaceae*
14.1	*Bacillus*	*Bacillaceae*
19.1.4	*Pseudomonas*	*Pseudomonadaceae*
10.4	*Lysinibacillus*	*Bacillaceae*
10.6	*Lysinibacillus*	*Bacillaceae*

[a]**Red designates isolates used in SC2 but not in SC1.**

Supplementary Table 3. Effects of *Enterobacter* pre-exposure on worm resistance to *E. faecalis*

Strain	[a]TD50 (N) following *E. coli* exposure	TD50 (N) following *CEent1* exposure	P-value, Logrank test	P-value, [c]Wilcoxon test
[d]*wt*<br>*dbl-1(nk3)*	137 (99)<br>158(62)	180 (93)<br>147 (59)	0.0092<br>0.0512	NS<br>NS
wt<br>*dbl-1(nk3)*	127 (96)<br>96 (83)	146 (77)<br>91 (90)	NS<br>NS	0.0283<br>NS
wt<br>*dbl-1(nk3)*	133 (103)<br>117 (102)	162 (97)<br>84 (103)	NS<br>0.0004	NS<br>P<0.0001
wt<br>*dbl-1(nk3)*<br>*dbl-1 o/e*	119 (96)<br>104 (89)<br>213 (94)	188 (90)<br>75 (83)<br>188 (102)	NS<br><0.0001<br>NS	0.01<br>0.0003<br>0.053
wt<br>*sma-3(e491)*<br>[e]*sma-3(wk30)*	115 (76)/120<br>187(78)/241<br>223(63)/267	148(96)/171<br>69(102)/100<br>64(77)/72	NS<br>P<0.0001<br>P<0.0001	0.054<br>P<0.0001<br>P<0.0001
wt<br>*dbl-1 o/e*<br>*sma-3(e491)*<br>*sma-3(wk30)*	97(99)/124<br>181(110)/195<br>95(99)/120<br>285(66)/404	150(130)/169<br>120(117)/140<br>78(117)/91<br>46(77)/68	<0.0001<br>0.0021<br>0.03<br>P<0.0001	<0.0001<br><0.0001<br>0.005<br>P<0.0001
wt<br>*dbl-1 o/e*<br>*sma-3(wk30)*	108(92)/120<br>130(100)/145<br>109(77)/145	137(96)/167<br>153(112)/167<br>69(67)/75	0.0157<br>NS<br>P<0.0001	0.003<br>NS<br>P<0.0001

[a] Time to death (in hours), in which 50% of the worms are dead.
[b] Significance of survival difference between worms of the respective strain pre-exposed to *E. coli* or to *CEent1*.
[c]Wilcoxon test gives less weight to data points in the trailing end of the experiment, which are based on a small number of individuals. Almost significant p-values are shown in black.
[d] Each block represents one independent experiment. In black are experiments were worms developed on *E. coli* or on *CEent1*; blue represents experiments in which worms were exposed to *CEent1* for 4 hours only at the end of development (L4), prior to shifting them to pathogen plates (worms maintained on E. coli served as controls).
[e] the full genotype is *sma-3(wk30);him-5(e1490)*.

Supplementary Table 4

**qRT-PCR primers (annealing temperature 60°C)**	
pan-actin forward	5'-TCGGTATGGGACAGAAGGAC-3'
pan-actin reverse	5'-CATCCCAGTTGGTGACGATA-3'
clec-65 forward	5'-CCCGGTGGTGACTGTGAATA-3'
clec-65 reverse	5'-AGCTCATATTGTCGCTGGCA-3'
clec-52 forward	5'-GGTGATGTCTTCATTGGTCTTG-3'
clec-52 reverse	5'-TCATGTTGTTGGTTCTCCATC-3'
dod-24 forward	5'-ACCGAGCCAGGAGGTTATCT-3'
dod-24 reverse	5'-TCCCGATGTTGATTTTGACC-3'
spp-8 forward	5'-TGCAAATCGATGGTTCAAAA-3'
spp-8 reverse	5'-CGGAGGTGTCAGCCAAGTAT-3'
folt-2 forward	5'-CAGGTCACAAACTACACCCAAA-3'
folt-2 reverse	5'-ACTTCGGTGATACCATTGGG-3'
**Primers for PCR calibration and for qPCR**	
27f (full-length 16S) - calibration	5'-AGAGTTTGATCMTGGCTCAG-3'
1492r (full-length 16S)	5'-TACGGYTACCTTGTTACGACTT-3'
806f	5'-AGATACCCCGGTAGTCC-3'
895r	5'-CYGYACTCCCCAGGYG-3'
Ent_MB_F	5'-ACCTGAGCGTCAGTCTTTGTC-3'
Ent_MB_R	5'-GTAGCGGTGAAATGCGTAGAGA-3'
Pse435F[8]	5'-ACTTTAAGTTGGGAGGAAGGG-3'
Pse686R[8]	5'-ACACAGGAAATTCCACCACCC-3'
BacilliF[9]	5'-GCAGTAGGGAATCTTCCGC-3'
BacilliR[9]	5'-ACACTTAGCACTCATCGTTT-3'
Ent-Hsp60f	5'-AAGGTTAAAGCGGGTGAAGG-3'
Ent-Hsp60r	5'-CGCGTATTGCAGAGCAGAA-3'
Hsp60For (500-bp product) - calibration	5'-GGTAGAAGAAGGCGTGGTTGC-3'
Hsp60Rev (500-bp Hsp60 product)	5'-ATGCATTCGGTGGTGATCATCAG-3'
**Primers used to identify and quantify synthetic community members**	
806r, 895f	*Eubacteria*
Ent_MB_F, Ent_MB_R	*Enterobacteriaceae*
Pse435F, Pse686R	*Pseudomonas* strains
BacilliF, BacilliR	*Bacillus, Lysinibacillus, Paenbacillus*
Ent-Hsp60f, Ent-Hsp60r	*Enterobacter*

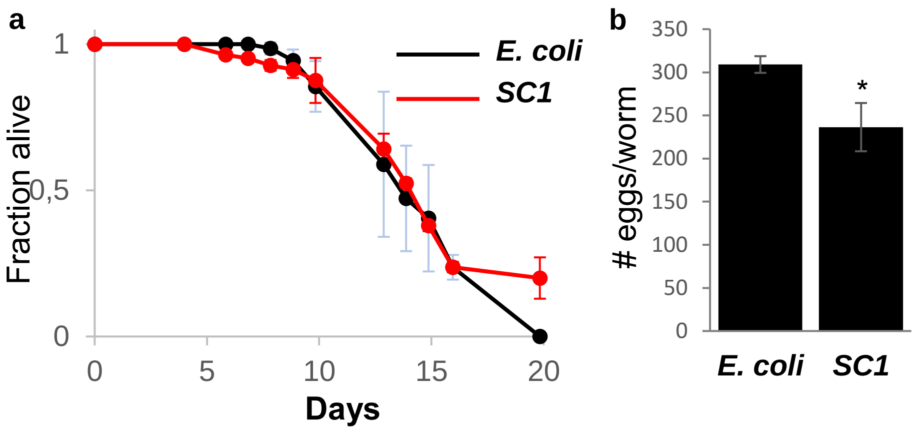

Supplementary Figure 1. Effects of growth on the SC1 synthetic community. (A) Worm lifespan on SC1 (at 25ºC) is similar to that of worms grown on *E. coli*, Shown are averages ± SD for experiments performed in duplicates (N=102-109 worms per group, NS, logrank test) from one experiment of two with similar results. (B) Egg laying. Shown is the total number of eggs laid over a period of 3 days (at 25°C). Averages ± SDs for five worms per group. *: p<0.001, t-test.

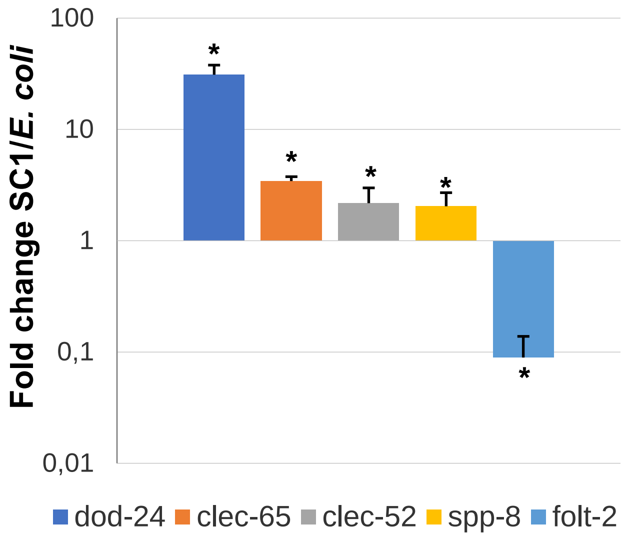

Supplementary Figure 2. Validation of RNAseq results. (A) Quantitative RT-PCR gene expression measurements for four genes upregulated in wildtype worms grown on SC1 (3 of which also upregulated in worms grown in soil microcosms), and one repressed gene, agree with RNAseq results. Shown are actin-normalized values, presented as fold change in worms raised on SC1 compared to those raised on *E. coli* OP50; averages ± SD for measurements performed in duplicates with RNA extracts from two independent experiments. Induction ranged from 2- to 31-fold. *: P<0.05, t-test.

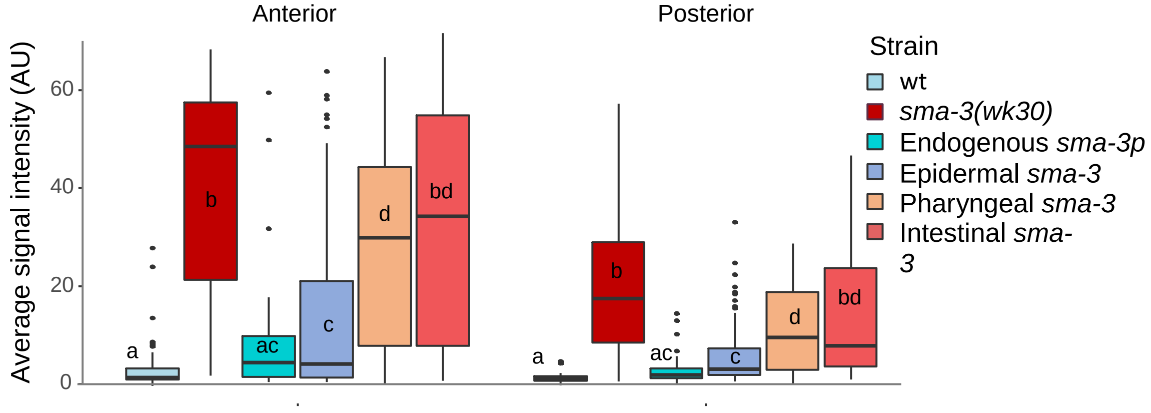

Supplementary Figure 3. TGFβ/BMP signaling controls *Enterobacter* colonization through multi-tissue contributions. Box plots quantifying fluorescent signal (normalized to area) in images as in Fig. 5A, in the anterior or posterior gut, in wildtype worms, *sma-3* mutants, or *sma-3* derivatives with transgenic tissue-specific *sma-3* expression, as designated. Statistically distinct groups are marked with letters (ANOVA and a Tukey's post hoc test; N=71(*wt*), 60(*sma-3 mutants*), 48(endogenous sma-3 promoter), 86 (epidermal *sma-3*), 40(pharyngeal *sma-3*), 33(intestinal *sma-3*)).

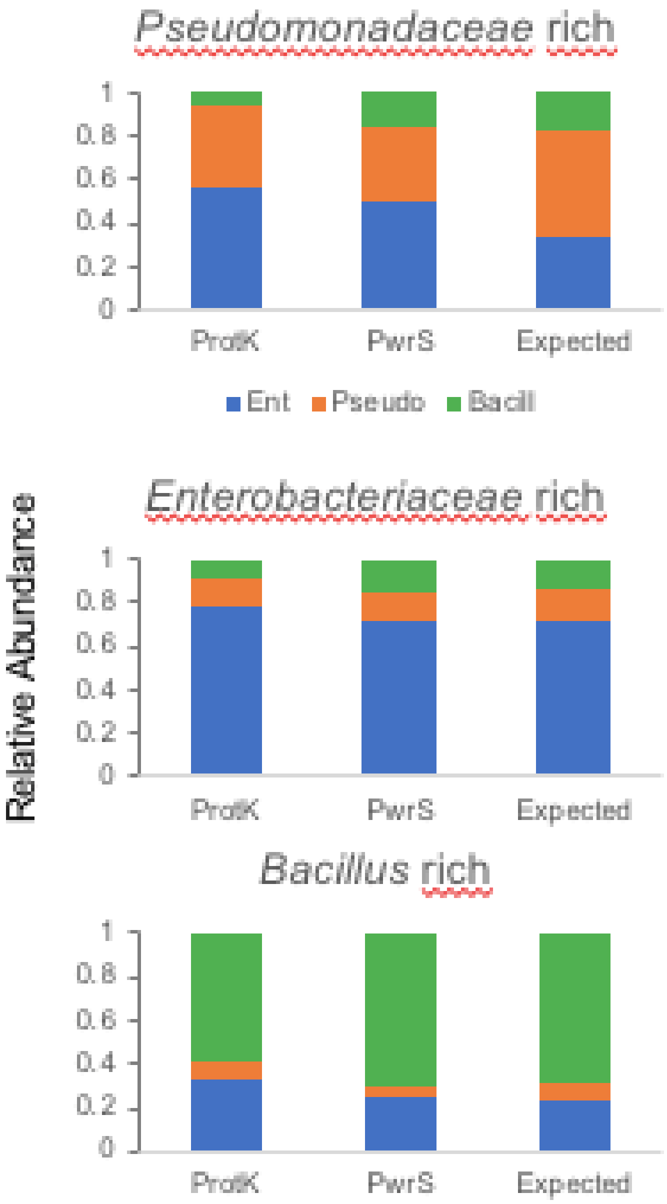

Supplementary Figure 4. Relative abundances measured via qPCR agree with calculated composition. Shown are **r**elative abundance values for bacteria of the designated families calculated based on taxa-specific qPCR measurements in DNA extracts from plate synthetic communities. DNA extraction was performed either with the protein K protocol used throughout the paper (ProtK), or with the MO-BIO PowerSoil DNA extraction kit (PwrS, following manufacturer instructions). Measurements from real DNA samples (N=2) are compared with the mix ratios used for preparation of the synthetic communities (Expected).

Supplementary References

1. Shapira, M. *et al.* A conserved role for a GATA transcription factor in regulating epithelial innate immune responses. *Proc Natl Acad Sci U S A* **103,** 14086–14091 (2006).
2. Roberts, A. F., Gumienny, T. L., Gleason, R. J., Wang, H. & Padgett, R. W. Regulation of genes affecting body size and innate immunity by the DBL-1 / BMP-like pathway in *Caenorhabditis elegans*. *BMC Dev Biol* **10,** 61 (2010).
3. Troemel, E. R. *et al.* p38 MAPK Regulates Expression of Immune Response Genes and Contributes to Longevity in *C . elegans*. *PLoS Genet* **2,** e183 (2006).
4. Murphy, C. T. *et al.* Genes that act downstream of DAF-16 to influence the lifespan of *Caenorhabditis elegans*. *Nature* **424,** 277–283 (2003).
5. Berg, M., Zhou, X. Y. & Shapira, M. Host-Specific Functional Significance of *Caenorhabditis* Gut Commensals. *Front. Microbiol.* **7,** 1622 (2016).
6. Montalvo-Katz, S., Huang, H., Appel, M. D., Berg, M. & Shapira, M. Association with soil bacteria enhances p38-dependent infection resistance in *Caenorhabditis elegans*. *Infect Immun* **81,** 514–520 (2013).
7. Berg, M. *et al.* Assembly of the *Caenorhabditis elegans* gut microbiota from diverse soil microbial environments. *ISME J.* **10,** 1998–2009 (2016).
8. Bergmark, L. *et al.* Assessment of the specificity of Burkholderia and Pseudomonas qPCR assays for detection of these genera in soil using 454 pyrosequencing. *FEMS Microbiol. Lett.* **333,** 77–84 (2012).
9. Hermann-Bank, M. L., Skovgaard, K., Stockmarr, A., Larsen, N. & Mølbak, L. The Gut Microbiotassay: A high-throughput qPCR approach combinable with next generation sequencing to study gut microbial diversity. *BMC Genomics* **14,** (2013).
