# Supplementary material for: TGFβ/BMP immune signaling affects abundance and function of C. elegans gut commensals
Source: Nat Commun. 2019 Feb 5;10:604. doi: 10.1038/s41467-019-08379-8 (PMC6363772; doi:10.1038/s41467-019-08379-8)
Supplement: Supplementary file 2 — Description of Additional Supplementary Files [file 41467_2019_8379_MOESM2_ESM.docx]

**Description of Additional Supplementary Files**

File Name: Supplementary Data 1

Description: List of genes differentially expressed during interactions with complex microbiotas.

File Name: Supplementary Data 2

Description: List of Gene Ontology annotations enriched among genes differentially expressed during interactions with complex microbiotas.
